# A Prospective Study of Sentinel Node Biopsy Omission in Women Age ≥ 65 Years with ER+ Breast Cancer

**DOI:** 10.1245/s10434-024-15000-w

**Published:** 2024-02-12

**Authors:** Alice P. Chung, Catherine M. Dang, Scott R. Karlan, Farin F. Amersi, Edward M. Phillips, Marissa K. Boyle, Yujie Cui, Armando E. Giuliano

**Affiliations:** 1 Department of Surgery, Cedars-Sinai Medical Center, Los Angeles, CA USA; 2 Department of Statistics, Cedars-Sinai Medical Center, Los Angeles, CA USA

**Keywords:** Breast cancer, Sentinel node biopsy, Age over 65, Estrogen-receptor positive, Breast conserving surgery

## Abstract

**Background:**

National guidelines recommend omitting SNB in older patients with favorable invasive breast cancer. However, there is a lack of prospective data specifically addressing this issue. This study evaluates recurrence and survival in estrogen receptor-positive/Her2− (ER+) breast cancer patients, aged ≥ 65 years who have breast-conserving surgery (BCS) without SNB.

**Methods:**

This is a prospective, observational study at a single institution where 125 patients aged ≥ 65 years with clinical T1-2N0 ER+ invasive breast cancer undergoing BCS were enrolled. Patients were treated with BCS without SNB. Primary outcome measure was axillary recurrence. Secondary outcome measures include recurrence-free survival (RFS), disease-free survival (DFS), breast cancer-specific survival (BCSS), and overall survival (OS).

**Results:**

From January 2016 to July 2022, 125 patients were enrolled with median follow-up of 36.7 months [95% confidence interval (CI) 35.0–38.0]. Median age was 77.0 years (range 65–93). Median tumor size was 1 cm (range 0.1–5.0). Most tumors were ductal (95/124, 77.0%), intermediate grade (60/116, 51.7%), and PR-positive (117/123, 91.7%). Radiation therapy was performed in 37 of 125 (29.6%). Only 60 of 125 (48.0%) who were recommended hormonal therapy were compliant at 2 years. Chemotherapy was administered to six of 125 (4.8%) patients. There were two of 125 (1.6%) axillary recurrences. Estimated 3-years rates of regional RFS, DFS, and OS were 98.2%, 91.2%, and 94.8%, respectively. Univariate Cox regression identified hormonal therapy noncompliance to be significantly associated with recurrence (*p* = 0.02).

**Conclusions:**

Axillary recurrence rates were extremely low in this cohort. These results provide prospective data to support omission of SNB in this patient population

**Trial Registration:**

ClinicalTrials.gov ID NCT02564848.

Breast cancer in women older than aged 65 years represents approximately 50% of breast cancer cases in the United States.^1^ Treatment in older patients presents unique challenges to providers who must optimize therapy while accounting for comorbidities, life expectancy, and effects of treatment on function. Older patients with early ER-positive/Her2− (ER+) breast cancer tend to have more favorable disease for which treatment de-escalation should be considered.

Although lymph node status remains an important prognostic indicator in breast cancer, tumor biology has become a stronger determinant of systemic therapy especially in postmenopausal women. Additionally numerous clinical trials have shown a lack of survival benefit for axillary dissection (ALND) versus no ALND in both node-positive and node-negative breast cancer.^2–9^

While sentinel node biopsy (SNB) has replaced ALND for surgical staging in clinically node negative breast cancer, the operation still is associated with some long-term morbidity, including limited range of motion, pain, paresthesias, and lymphedema.^10–16^ In addition, routine use of SNB in this patient population has not been shown to be cost effective.^17^

Prospective data demonstrating a lack of benefit from ALND and retrospective data demonstrating low recurrence rates when SNB is omitted in older women led to a recommendation for SNB omission in the Society of Surgical Oncology Choosing Wisely campaign.^18–20^ The relevant guidelines state: “Do not routinely perform sentinel node biopsy in clinically node-negative women over age 70 with early-stage hormone receptor positive, Her2− breast cancer.” However, there is a lack of prospective data specifically evaluating omission of SNB to support this recommendation.

This study is a prospective, observational study of women aged 65 years and older with early ER+ invasive breast cancer who were treated with BCS with the purpose of evaluating the safety and efficacy of SNB omission. We hypothesized that this approach would not impact survival in this patient population.

## Methods

From January 2016 through July 2022, patients were enrolled in this trial if they were at least age 70 years, had a clinical T1-2N0, ER+, Her2− invasive breast cancer without suspicious findings on preoperative axillary ultrasound and were planning to undergo BCS with adjuvant hormonal therapy with or without radiation. Informed consent was obtained from eligible participants and all trial related activities were performed after approval by the local Human Investigations Committee and in accord with the institution’s guidelines for experimental investigation of human subjects. The age criterion was changed to include patients aged 65 years and older in January 2017. Exclusion criteria comprised of younger than aged 65 years, clinical T3 or T4 tumors, palpable lymph nodes, biopsy proven lymph node metastases, ER− or Her2+ breast cancer, history of previous ipsilateral breast cancer, and plan to undergo mastectomy. The study was presented to patients during initial surgical consultation before the operation in patients with a known cancer diagnosis established by preoperative core needle biopsy. In cases where invasive cancer was diagnosed by a surgical biopsy, eligible patients were informed about the study during the visit preceding BCS. Patients could be enrolled to the trial preoperatively or up to 60 days postoperatively. Lumpectomy was performed to negative margins with reexcision if necessary. Patients were then followed on a semiannual basis with mammogram and clinical breast exam for 2 years and then annually.

Data regarding patient and tumor characteristics, treatment, and follow-up status was obtained. The following disease processes were included as comorbidities, based on the National Cancer Institute Comorbidity Index: liver disease, cerebrovascular disease, peripheral vascular disease, renal disease, dementia, heart disease, diabetes, pulmonary disease, blood disorders, immune disease, and other malignancy. The primary endpoint was regional recurrence. Secondary endpoints included local recurrence, disease-free survival (DFS), breast cancer-specific survival (BCSS), and overall survival (OS). Target accrual was 150. Interim analysis was performed after enrolling 125 patients with median follow-up of 36.7 months.

Patient demographic and clinical characteristics were summarized by using means and standard deviations for continuous variables, and frequencies and percentages for categorical variables, respectively. The Kaplan–Meier method was used to estimate the median survival time for all survival endpoints. Median follow-up time was estimated using the reverse Kaplan–Meier method. Univariate Cox proportional hazard regression was used to identify potential risk factors for regional recurrence, DFS, or OS. Significance level for two tailed hypothesis tests were set at 0.05. All statistical analyses were conducted by using statistical software R (R Core Team, v4.2.1).

## Results

**Fig. 1:**
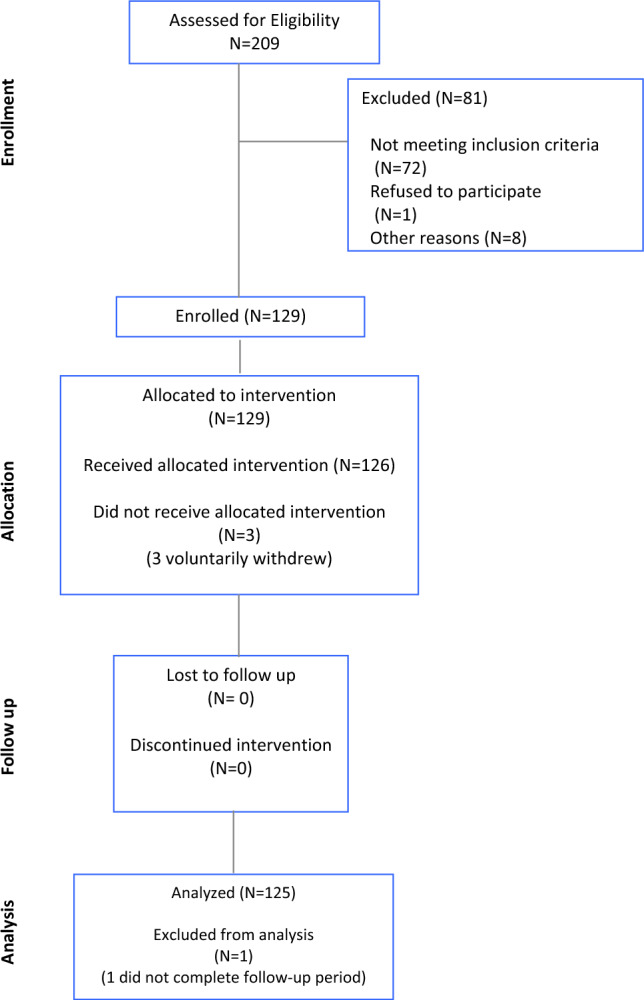
Consort diagram

During the study period, 209 patients were screened, and 125 patients who met eligibility criteria were enrolled (Fig. 1). Median tumor size was 1 cm (range 0.1–5). Most tumors were T1c (42/124, 33.8%), intermediate grade (60/116, 51.7%), had ki67 ranging from 6 to 20% (62%), and were ductal in origin (95/124, 76.6%) (Table 1). Nearly all tumors had PR positivity (117/123, 95.1%) and absence of lymphovascular invasion (111/121, 91.7%). Among the tumors where oncotype recurrence score was evaluated (*N* = 28), the median recurrence score was 16.^3,32^

**Table 1:** Tumor characteristics

Characteristics	*N* (%)
*Tumor stage*	*N* = 124
Tmic	4 (3%)
T1a	19 (15%)
T1b	31 (25%)
T1c	42 (34%)
T2	28 (23%)
*Tumor grade*	*N* = 116
I	42 (36%)
II	60 (52%)
III	14 (12%)
*Ki67*	*N* = 116
< 5%	28 (24%)
6–20%	72 (62%)
> 20%	16 (14%)
*Histology*	*N* = 124
Ductal	95 (77%)
Lobular	14 (11%)
Mixed or other	15 (12%)
*Lymphovascular invasion*	*N* = 121
Absent	111 (92%)
Present	10 (8%)

The median age was 77 (range 65–93) years. Eighty-six percent of patients (107/125) had at least one comorbidity, and 46.4% (58/125) had more than two comorbidities. Radiation therapy was performed in 37 of 125 (29.6%): 3 of 37 (8.1%) had accelerated partial breast irradiation; 34 of 37 (91.9%) had whole breast radiation. Patients were recommended to take hormonal therapy, and compliance was relatively poor. Only 60 of 125 (48.0%) patients were compliant with hormonal therapy at or beyond 2-years follow-up. At 3 years after the operation, only 30% were on hormonal therapy. Twenty-nine (23.2%) patients did not take any hormonal therapy at all. Chemotherapy was administered to 6 of 125 (4.8%) patients (Table 2).

**Table 2:** Patient and treatment characteristics (*N* = 125)

Comorbidities	
0	18 (14.4%)
1	21 (16.8%)
2	28 (22%)
> 2	58 (46%)
Radiation	37 (30%)
Chemotherapy	6 (5%)
Hormonal therapy compliance	
≥ 2 years	60 (48%)
≥ 3 years	37 (30%)
Not at all	29 (23%)

At median follow up of 36.7 months (95% CI, 35.0–38.0), there were six (4.8%) local recurrences and two (1.6%) axillary recurrences. Those who developed axillary recurrences had a concurrent distant recurrence. There was one tumor recurrence at 18 months of a poorly differentiated multifocal invasive lobular carcinoma, ER+, PR+, Her2−, and ki67 of 20%, with two foci (4.5 cm and 1.5 cm). The patient received hormonal therapy but declined breast radiation. The other patient whose tumor recurred regionally at 12 months also had a multifocal grade 2 invasive lobular carcinoma, ER+, PR+, Her2−, ki67 32%, with the largest tumor focus of 3.5 cm. She had a significant delay in seeking medical attention, and after her tumor resection declined all adjuvant therapy. This second patient eventually died of metastatic breast cancer and was the only patient who died of breast cancer in the cohort. One other patient developed a distant recurrence at 60 months of follow-up.

**Fig. 2:**
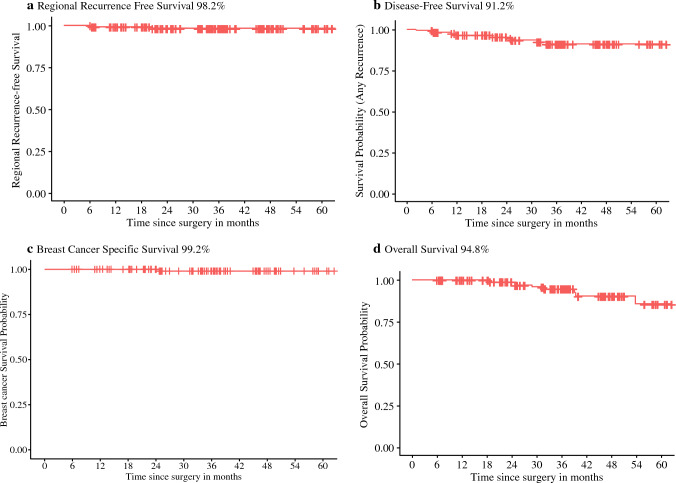
Kaplan–Meier curves of estimated 3-year outcomes for regional recurrence-free survival (**a**), disease-free survival (**b**), breast cancer-specific survival (**c**), and overall survival (**d**)

The estimated 3-years rates of regional recurrence-free survival, DFS, BCSS, and OS were 98.2%, 91.2%, 99.2%, and 94.8%, respectively (Fig. 2). Univariate cox regression analyses did not identify any factors to be significantly associated with local recurrence, regional recurrence, DFS, or OS (Table 3). Lymphovascular invasion and receipt of adjuvant chemotherapy and radiation therapy were not reported in the table, because these variables were associated with hazard ratios of 0 and wide confidence intervals because of a complete lack of events in the reference group. Hormonal therapy noncompliance was the only factory significantly associated with any recurrence (*p* = 0.02). Cardiovascular disease was the primary cause of death (3/8, 37.5%) (Table 4). There was one death from breast cancer in the patient described previously.

**Table 3:** Univariate analysis for any recurrence

Characteristic	*N*	Hazard ratio	95% CI	*p*
Age	125	1.10	0.99–1.21	0.08
Race (Caucasian vs. ref African American)	125	0.33	0.07–1.60	0.20
Ethnicity (non-Hispanic vs. ref Hispanic)	124	0.27	0.03–2.17	0.20
Body mass index	125	1.01	0.9–1.12	0.90
Comorbidities	125	1.15	0.14–9.18	0.9
Tumor grade	116			
Grade 2		5.15	0.63–41.90	0.13
Grade 3		3.29	0.21–52.6	0.4
PR-positivity	123	0.42	0.05–3.38	0.40
Histology	124			
Lobular (vs. ref ductal)		2.31	0.48–11.20	0.30
Tumor size	125	1.58	0.92–2.69	0.095
Ki67 (ref ≤ 5%)	116			
6–19%	116	2.38	0.29–19.8	0.4
≥ 20%	116	3.49	0.32–38.5	0.3
Hormonal therapy compliance < 2 years (vs. ref of compliance ≥ 2 years)	125	2.60	0.36–18.6	0.3
Hormonal therapy noncompliance (vs. ref of compliance ≥ 2 years)	125	6.34	1.23–32.8	0.028

*CI* confidence interval, *ref* reference group, *PR* progesterone receptor

**Table 4:** Causes of death

Cause of death	Time of last follow-up (months)
Cardiovascular	36
Cardiovascular	48
Cardiovascular	18
Aplastic anemia	24
Multiple comorbidities	36
Natural cause	18
Ureteral cancer	36
Breast cancer	36

## Discussion

Elderly patients with invasive breast cancer tend to present with favorable disease, so the need for SNB has been questioned. Various studies have demonstrated a lack of survival advantage in older women with early ER+ invasive breast cancer who have axillary surgical staging.^21–24^ Whereas several randomized, controlled trials have demonstrated no benefit of axillary dissection compared with no axillary dissection, there is a paucity of prospective data addressing the safety of SNB omission. This prospective, observational study reports outcomes in women aged 65 years and older with ER+ invasive breast cancer undergoing BCS without axillary SNB and demonstrates very few events associated with SNB omission.

There were only two axillary recurrences, both associated with distant recurrence. Only one death was attributed to breast cancer. Most deaths were cardiovascular in nature. The median patient age was 77%, and 86% of patients had at least one comorbidity; 46% of patients had more than two comorbidities. Studies of women older than aged 65 years with breast cancer have shown that approximately 45–50% of patients have comorbidities,^25^ and as age increases, comorbidity rate increases.^26^ The higher rate of comorbidities and death from causes other than breast cancer support the case for deescalating therapy in this cohort.

There were only two regional recurrences in the group, both of which occurred in patients older than aged 80 years. Unfortunately, these recurrences were associated with concurrent distant recurrences. Specific data on these patients revealed that their tumors were lobular, multifocal, and had high ki67. One patient also had a significant delay in operative intervention and declined all adjuvant therapy. The other patient declined radiation but took hormonal therapy. It is possible that patients with these high-risk tumor characteristics might benefit from axillary surgical staging, although it is unclear whether that intervention would have prevented regional or distant recurrence. Furthermore, these tumor factors were not shown to be significant predictors of recurrence in univariate analysis. These findings corroborate the results of retrospective data that has been published on SNB omission in older patients as well as the randomized, controlled trials evaluating the lack of benefit of axillary dissection in older patients. In a previous retrospective analysis of 140 patients older than aged 70 years with ER+ disease from our institution who did not have SNB, only one patient developed an axillary recurrence with 4.5-years median follow-up.^19^ The randomized trial of outcomes in elderly patients with T1N0 breast cancer who had ALND compared with those who did not have ALND found that four of 110 (3.6%) patients who did not have ALND developed axillary recurrence 7–157 months after surgery.^5^ The randomized IBCSG 10-93 trial compared 473 women older than aged 60 years with clinically node-negative operable breast cancer who had ALND versus those who had no ALND.^7^ They found that after median follow-up of 6 years, axillary recurrence rates were extremely low in both arms (1.0% vs 2.5%) with no difference in DFS or OS between arms. The regional recurrence rate in the current study was 1.6% with median follow-up of 3 years. This was an interim analysis without all planned patients included and with only half of the anticipated follow-up achieved. More recurrences are likely to be expected. However, previous studies have shown that 3-years follow-up is likely to capture the majority of regional recurrences.^2,4,5^

Univariable analysis identified noncompliance with hormonal therapy to be the only factor to be associated with any recurrence. No other patient or tumor factors, including SNB or radiation therapy, were associated with recurrence. SNB has not been shown in retrospective studies to be associated with local, regional recurrence, or OS.^27–29^ Sun and colleagues conducted a retrospective review of 500 patients older than aged 70 years and did not find an association between SNB and recurrence but found that those who did not complete hormonal therapy had worse OS and DFS.^28^ Carleton et al. studied 487 women older than aged 70 years treated with BCS and found that SNB was not associated with recurrence and radiotherapy was the only factor associated with reduced recurrence with median follow-up of 4.8 years.^27^ McKevitt et al. evaluated 2662 patients older than aged 70 years with clinically node-negative HR+ breast cancer treated with SNB.^30^ The authors found that with a median follow-up of 4.3 years, BCSS was worse with higher-grade tumors and was improved by use of hormonal therapy. They also found that BCSS was similar in patients with a metastatic sentinel node versus those who had a benign SNB when patients received hormonal therapy, demonstrating the value of hormonal therapy in reducing recurrence. They concluded that SNB can be safely omitted in older patients who take hormonal therapy. Poor compliance with hormonal therapy has been shown to be associated with worse survival.^31^ In the present study, compliance with hormonal therapy was poor, and poor compliance was associated with any recurrence.

Challenges with de-implementation of SNB have been observed. Despite the publication recommendations in the 2016 Choosing Wisely Campaign to avoid SNB in older patients with favorable ER+ breast cancer, 65–80% of patients who meet guideline criteria still have SNB performed.^27,29,32^ Barriers to de-implementation have been reported to be associated with patient fear, malpractice concerns, desire for more scientific data, physician preference because of lack of familiarity or skepticism toward the recommendations.^33,34^ As more prospective data matures, there may be greater adoption of de-implementation of axillary surgical staging. One challenge with de-implementation of SNB includes concerns of oncologists that patients will not comply with hormonal therapy and thus be at higher risk for recurrence. Despite the low compliance rate in our study, outcomes remained excellent, although longer follow-up is needed.

Utilization of adjuvant radiation therapy was equally low; less than 30% of patients received radiation therapy. Another challenge in de-escalating axillary surgery in older patients is the concern that omission of both SNB and radiation therapy may be undertreatment. Dahn et al. evaluated 460 patients older than aged 70 years with T1N0 breast cancer and found that those who did not receive adjuvant therapy had worse 5-year local recurrence-free, locoregional recurrence-free, and DFS than those receiving at least one form of adjuvant therapy. In this study, compliance with hormonal therapy was poor, and poor compliance was associated with any recurrence.^31^ However, in the CALGB 9343 trial, 37% of patients did not have axillary surgical staging. The study follow-up of more than 10 years demonstrated no difference in survival.^21,22^ The PRIME II trial evaluated safety of radiation omission in patients older than aged 65 years.^35,36^ The investigators found that only 30% of patients had a SNB, and no difference in survival was demonstrated with 10-years follow-up. Although neither the CALGB 9343 or PRIME II trial was designed to evaluate safety of SNB and radiation omission, the subgroup analyses showed that concurrent de-implementation of both therapies did not negatively impact outcomes.

There was a significant proportion of patients in this study who elected to omit both radiation and endocrine therapy. Despite this, recurrence rates remained low, although longer follow-up is likely to reveal more recurrences. This highlights the need for multidisciplinary discussion with patients regarding patient preferences and tolerability of adjuvant therapy.

There may be little benefit to SNB in older patients with clinical T1-2N0 ER+ breast cancer undergoing BCS. It is possible that women treated with mastectomy may not derive significant benefit from axillary surgical staging. We chose to exclude patients undergoing mastectomy to minimize variations in surgical treatment.

Although this was a prospective, clinical trial, there may have been selection bias in enrolling patients. Most patients were older and had multiple comorbidities and smaller tumors with low or intermediate grade. Whereas patients with younger age, higher grade, and larger tumor size were eligible, they only represented a very small percentage of cases in this cohort. These data, along with retrospectively published data, clearly support the omission of SNB in older patients with early ER+ disease. For younger patients with larger tumors, de-implementation of axillary surgical staging is still unlikely to be adopted until larger studies with longer follow-up in those subgroups are performed. Additionally, with the recent approval of Abemaciclib for the treatment of early high-risk ER+ breast cancer, sentinel node status might be valuable in identifying candidates for this CDK4/6 inhibitor. One may consider performing SNB in patients with early ER+ breast cancer if the tumor has a high grade or high ki67.

The safety of SNB omission is currently under investigation in other patient populations. The Sound trial randomized more than 1400 patients aged > 45 years with similar tumor characteristics to SNB versus no SNB.^37^ Cyr and colleagues reported results of their pilot randomized trial comparing SNB to no SNB in clinical T1-2N0 breast cancer patients with benign axillary ultrasound.^38^ After median of 17 months of follow-up, there were no axillary recurrences in either arm; the negative predictive value of axillary ultrasound was 96.9%. Omission of SNB is even being considered in higher risk subtypes of breast cancer. Zhong et al. reported that outcomes in older patients with triple-negative and Her2+ breast cancer who did not have SNB were similar to those with luminal-type breast cancer, suggesting that omission of SNB could be considered for more aggressive subtypes of breast cancer.^39^ With the accumulation of more prospective data in this population and higher-risk subgroups, one would expect to see increase in the de-implementation of axillary surgical staging.

## Conclusions

Despite low utilization of adjuvant therapy, outcomes of SNB omission are excellent, with axillary recurrence rate of 1.6% at 3 years. Women older than aged 65 years with clinical T1-2N0 ER+ breast cancer undergoing BCS are unlikely to gain benefit from SNB. The preliminary results of this trial provide support for de-escalating axillary surgical staging in this patient population.

## Disclosure

This research was supported by The Fashion Footwear Charitable Foundation of New York, Inc., The Margie and Robert E Petersen Foundation, and Linda and Jim Lippman. The supporters had no role in the data collection, analysis, interpretation, writing of the manuscript, or decision to submit.
